# MCPIP1 Deficiency in Mice Results in Severe Anemia Related to Autoimmune Mechanisms

**DOI:** 10.1371/journal.pone.0082542

**Published:** 2013-12-06

**Authors:** Zhou Zhou, Ruidong Miao, Shengping Huang, Brandon Elder, Tim Quinn, Christopher J. Papasian, Jifeng Zhang, Daping Fan, Y. Eugene Chen, Mingui Fu

**Affiliations:** 1 Cardiovascular Center, Department of Internal Medicine, University of Michigan Medical Center, Ann Arbor, Michigan, United States of America; 2 Department of Basic Medical Science, School of Medicine, University of Missouri Kansas City, Kansas City, Missouri, United States of America; 3 Department of Cell Biology and Anatomy, University of South Carolina School of Medicine, Columbia, South Carolina, United State of America; Northwestern University Feinberg School of Medicine, United States of America

## Abstract

Autoimmune gastritis is an organ-specific autoimmune disease of the stomach associated with pernicious anemia. The previous work from us and other groups identified MCPIP1 as an essential factor controlling inflammation and immune homeostasis. MCPIP1^-/-^ developed severe anemia. However, the mechanisms underlying this phenotype remain unclear. In the present study, we found that MCPIP1 deficiency in mice resulted in severe anemia related to autoimmune mechanisms. Although MCPIP1 deficiency did not affect erythropoiesis *per se*, the erythropoiesis in MCPIP1^-/-^ bone marrow erythroblasts was significantly attenuated due to iron and vitamin B_12_ (VB_12_) deficiency, which was mainly resulted from autoimmunity-associated gastritis and parietal cell loss. Consistently, exogenous supplement of iron and VB_12_ greatly improved the anemia phenotype of MCPIP1^-/-^ mice. Finally, we have evidence suggesting that autoimmune hemolysis may also contribute to anemia phenotype of MCPIP1^-/-^ mice. Taken together, our study suggests that MCPIP1 deficiency in mice leads to the development of autoimmune gastritis and pernicious anemia. Thus, MCPIP1^-/-^ mice may be a good mouse model for investigating the pathogenesis of pernicious anemia and testing the efficacy of some potential drugs for treatment of this disease.

## Introduction

 Anemia is a group of red blood cell (RBC) dysfunction or loss related diseases, which is caused by many reasons and mechanisms. Autoimmune gastritis is an organ-specific disease that is the underlying pathologic cause of vitamin B_12_ deficiency, the most common cause of pernicious anemia. The pathogenesis of autoimmune gastritis and its associated pernicious anemia is not completely understood due to lack of optimal animal models for this disease.

 RBCs are continuously produced from bone marrow by differentiation and proliferation of hematopoietic stem cells (HSCs) [1]. Destroying the HSCs would result in aplastic anemia [2]. In addition, normal function of other organs is also important for RBC production and survival. For example, kidneys can secrete erythropoietin (EPO) and EPO is a critical hormone that drives RBC differentiation [3]. The parietal cells of stomach express internal factor to facilitate VB_12_ absorption. VB_12_ deficiency would cause pernicious anemia [4]. The acid environment of stomach and normal function of intestine are critical for the absorption of iron, a necessary element in oxygen carrier protein hemoglobin in RBCs [5]. Liver secretes transferrin to carry iron from intestine and other organs to bone marrow to facilitate RBC production [6]. Liver also secretes hepcidin to lower the absorption and usage of iron to regulate iron level in the body [6]. 

 The homeostasis of immune system is also important for maintaining the normal life span of RBCs. First, old and damaged RBCs are normally engulfed by macrophages and then iron can be recycled [7]. Second, chronic inflammation and high interleukin-6 (IL-6) level would trigger hepcidin expression, which is the only hormone dedicated in regulating iron metabolism in mammals. Increased hepcidin inhibits iron absorption in intestine and increase iron store in the macrophages of spleen and liver, and lowers the plasma iron concentration. This would finally cause inflammation related anemia [6]. Third, some types of anemia are driven by autoimmunity against critical organs or cells responsible for the normal RBC homeostasis. Autoimmunity against the parietal cells in stomach mucosa can result in pernicious anemia featuring VB_12_ deficiency and possibly accessory iron deficiency [4]. Also, anti-RBC autoimmune antibodies would damage the RBCs and speed their clearance, resulting in decreased RBC number and anemia. 

 MCP-1 induced protein 1 (MCPIP1) is a recently discovered protein, which is essential for regulating inflammatory response and immune homeostasis [8,9]. We and others have found that MCPIP1^-/-^ mice showed growth retardation and premature death [8,9]. These mice developed global inflammation with leukocyte infiltration in multiple organs and expanded spleens and lymph nodes. A series of work demonstrated that MCPIP1 could modulate NF-κB signal transduction and the mRNA stability of inflammatory cytokines such as IL-6, IL-2 and IL-12. However, these mechanisms could not totally explain the detrimental phenotypes of MCPIP1^-/-^ mice. In the present study, we found the deficiency of MCPIP1 caused severe anemia that was resulted from iron deficiency, VB_12_ deficiency and RBC destroy. The MCPIP1 deficient mice spontaneous developed autoimmunity against parietal cells of the stomach mucosa, which compromised the absorption of VB_12_ and iron. The iron and VB_12_ deficiency resulted in reduced erythroblast proliferation and increased apoptosis, which finally caused anemia. Exogenous supplement of iron and VB_12_ greatly improved the anemia phenotype of MCPIP1^-/-^ mice. In addition, MCPIP1^-/-^ mice also developed RBC autoimmune antibody to accelerate the RBC clearance and devastate the anemia phenotype. Thus, the MCPIP1 deficient mice may be a novel animal model of autoimmune gastritis and consequent pernicious anemia. 

## Materials and Methods

### Mice

 6 weeks old MCPIP1^-/-^ mice and littermate controls in C57BL/6 background were bred and housed in the Laboratory Research Animal Center of University of Missouri Kansas City under specific pathogen-free conditions. For iron and VB_12_ supplementation, 250 μg/g body weight iron dextrin and/or 250 μg VB_12_ (both from Sigma Aldrich) were subcutaneously injected, and further analyzed 7 days later. Experimental procedures were approved by the Animal Care and Use Committee of University of Missouri Kansas City. 

### Flow cytometry

 The MCPIP1^-/-^ bone marrow cells were collected from both femurs and tibias. Bone marrow cells and splenocytes were then stained with fluorescence conjugated antibodies against membrane antigens. If an intracellular antigen or DNA was detected, the cells were fixed, permeabilized and then stained with the antibodies (Foxp3 staining kit from eBioscience). The staining patterns were then detected with FACS Canto flow cytometry device and then further analyzed with FlowJo (TreeStar). 

 The antibodies used in this work include anit-Ter119-PE, anti-CD71-FITC, anti-IgG-FITC, anti-IgM-FITC, anti-IgA-FITC (all from eBioscience) and anti-cleaved caspase-3-Alexa Fluor-647 (BD bioscience). The DNA was stained with 7-AAD (eBioscience). The reticulocyte percentage was determined with Reti-COUNT (thiazole orange) reagent (BD Biosciences). 

### Histochemistry

 For the morphological analysis of liver, spleen, bone and stomach, the organs were collected and then fixed with 4% paraformaldehyde. After that, the slides were prepared and then stained with hematoxylin and eosin for H.E. staining or with iron staining kit for Prussian blue staining, following the instructions of the manufacturer. For the blood cell analysis, peripheral blood was collected and single layer blood smear was prepared, followed by Giemsa-Wright staining following the manufacturer’s instructions. All of the above dyeing chemicals were purchased from Sigma Aldrich. The immunofluorescence staining involved DyLight 594 anti-mouse IgG (Vector Laboratories). 

### RNA isolation, reverse transcription and real-time PCR

 The livers and intestines were collected with Trizol (Invitrogen). The RNA was purified following the instructions of the manufacturer. The first strand of cDNA was synthesized with the High Capacity cDNA Reverse-transcription Kit. The real-time PCR was performed by using SYBR Green PCR master mix system (both from Applied Biosystems). The primers were listed in Table 1. 

**Table 1:** Primers used in the real-time PCR analysis.

Gene	Primer	sequence
HO-1	Upper	GCCACCAAGGAGGTACACAT
	Lower	GTGGAGACGCTTTACATAGTGC
HO-2	Upper	CTACTCAGCCACAATGTCTTCA
	Lower	TGGTCCTTGTTGCGGTCCAT
Ferritin H	Upper	CAACAGTGCTTGAACGGAACC
	Lower	CTTCAGAGCCACATCATCTCG
Ferritin L	Upper	CGTGGATCTGTGTCTTGCTTCA
	Lower	AAGTTGACCAGGCGGTTCAC
Transferrin	Upper	AATGGGCTAAGAATCTGAAGCA
	Lower	TACCCTCTGGAAGTTTAACGAA
DMT-1	Upper	GTGGTTAGCGTGGCTTATTTGA
	Lower	AGGTCTCCTGGGCTGTTAGT
Ferriportin	Upper	TACAGGGTACGCCTACACTCA
	Lower	CATTCACAAACCTAGAACGGATA
IRP-1	Upper	GGACATCGTGCTCACCATTAC
	Lower	TGCCTACAGCCTGAAGATACTT
IRP-2	Upper	TAGGGTTTGACATAGTTGGCTA
	Lower	GGGCGGAGAGGCGAGATAAT
Hepcidin	Upper	CAGGGCAGACATTGCGATAC
	Lower	TTATTTCAAGGTCATTGGTGGG
BMP-6	Upper	CTTGTGGTTGCCATTAAGTTGA
	Lower	GAGGCGAACATTAGGTAACTG
β-actin	Upper	GGCTACAGCTTACCACCAGG
	Lower	GCCAGACAGCAGTGTGTTGGC

### ELISA assay

 Mouse peripheral blood plasma was collected from 6 weeks old MCPIP1^-/-^ mice and littermate controls. The EPO and IL-6 concentration was examined with EPO and IL-6 detection ELISA kit (BD biosciences) respectively following the manufacturer’s instructions. 

### In vitro colony forming assay

 Complete methylcellulose medium (Methocult M3434, StemCell Technologies) was used to evaluate the colony forming potentials according to the manufacturer’s instructions. 2×10^5^ spleen cells or bone marrow cells were used in each sample. All the tests were performed in duplicate. The CFU-es were counted 2 days after the culture and the BFU-es were counted 7 days after the culture. 

### Plasma total iron concentration assay

 Plasma was prepared by collecting the blood from the retro-orbital bleeding with anti-coagulation of heparin, and then centrifuged immediately. The total iron detection kit (ThermoDMA) was used to determine the total plasma iron concentration, following the instructions of the manufacturer. 

### Statistics

 All the original data shown represent one of at least three independent experiments. To compare two groups, unpaired *t*-test was used. Data were presented as mean±SEM. P<0.05 was considered as statistically significant. 

## Results

### MCPIP1^-/-^ mice developed severe anemia

**Figure 1:**
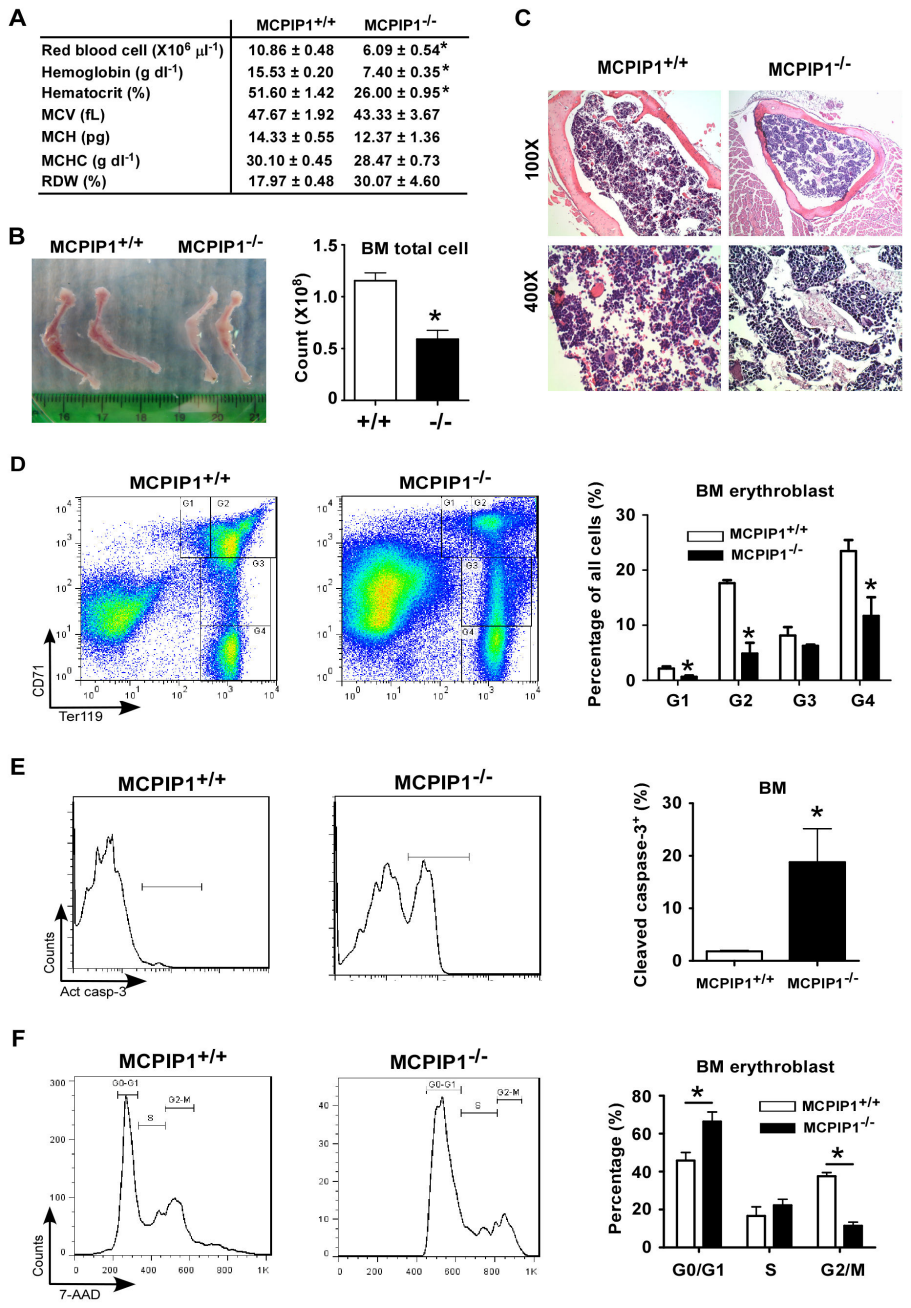
MCPIP1^-/-^ mice developed severe anemia. Peripheral red blood cell count was performed on 6 weeks old MCPIP1^+/+^ and MCPIP1^-/-^ mice (A). The hind limb bones were shown and the total bone marrow cells from both femurs and tibias were further counted (B). The femur bone marrow was also stained with H.E. (C). The bone marrow cells were stained with Ter119 and CD71, and gated to G1~G4. The percentages of these gates were compared between the MCPIP1^+/+^ and MCPIP1^-/-^ mice (D). The Ter119 and CD71 double positive cells were further stained with active caspase-3 (E) and 7-AAD (F). The active caspase-3^+^ cells and the cell cycle stages were statically analyzed. N=5~6. *P<0.05.

 Previous work from us and others suggested that MCPIP1 deficiency in mice resulted in anemia [8,9]. To further determine the anemia phenotype in MCPIP1^-/-^ mice, we performed peripheral blood count test in 6 weeks old male MCPIP1^-/-^ mice and littermate controls. MCPIP1^-/-^ mice showed significantly decreased RBC count, hematocrit and hemoglobin concentration compared with that in wild-type mice (Figure 1A). The mean corpuscular volume (MCV), mean corpuscular hemoglobin (MCH), mean corpuscular hemoglobin concentration (MCHC) and red blood cell distribution width (RDW) did not show significant changes (Figure 1A). These results confirmed that MCPIP1^-/-^ mice developed severe anemia. Since the anemia phenotype is featured by reduced RBC count, we next examined the bone marrow erythropoietic activity of MCPIP1^-/-^ mice. As shown in Figure 1B, the bone marrow of MCPIP1^-/-^ mice was paler than MCPIP1^+/+^ mice, and the total cell amount was also lower. The histological staining also showed reduced hemoglobin containing cells in the MCPIP1^-/-^ bone marrow (Figure 1C). To better quantify RBC producing cells, we examined the erythroblasts in the bone marrow by flow cytometry analysis, which were stained with CD71 and Ter119 antibodies and further gated into G1 ~ G4 (G1, proerythroblasts; G2, basophilic erythroblasts; G3, late basophilic and polychromatophilic erythroblasts and G4, orthochromatophilic erythroblasts) [8]. The MCPIP1^-/-^ bone marrow contained significant lower percentages of the early G1 and G2 phase erythroblasts as well as late G4 phase (Figure 1D), which indicated a defect in MCPIP1^-/-^ bone marrow erythropoiesis. Taken together, these results suggested that the MCPIP1^-/-^ mice had severe anemia related to abnormality of erythropoiesis. 

 To further define the reason of the erythropoietic defect, we analyzed the viability and proliferation of the erythroblasts in MCPIP1^-/-^ mice. MCPIP1^-/-^ erythroblasts showed higher percentage of active caspase-3^+^ cells (Figure 1E), indicating an increased apoptosis of these cells comparing to MCPIP1^+/+^ cells. In addition, MCPIP1^-/-^ erythroblasts reside more in G0/G1 phase while less in G2/M phase (Figure 1F), suggesting a reduced capability of proliferation. Thus, the reduced viability and proliferation of erythroblasts might be the direct reason of anemia in MCPIP1^-/-^ mice. 

### MCPIP1^-/-^ BM showed increased erythropoietic capacity in vitro

**Figure 2:**
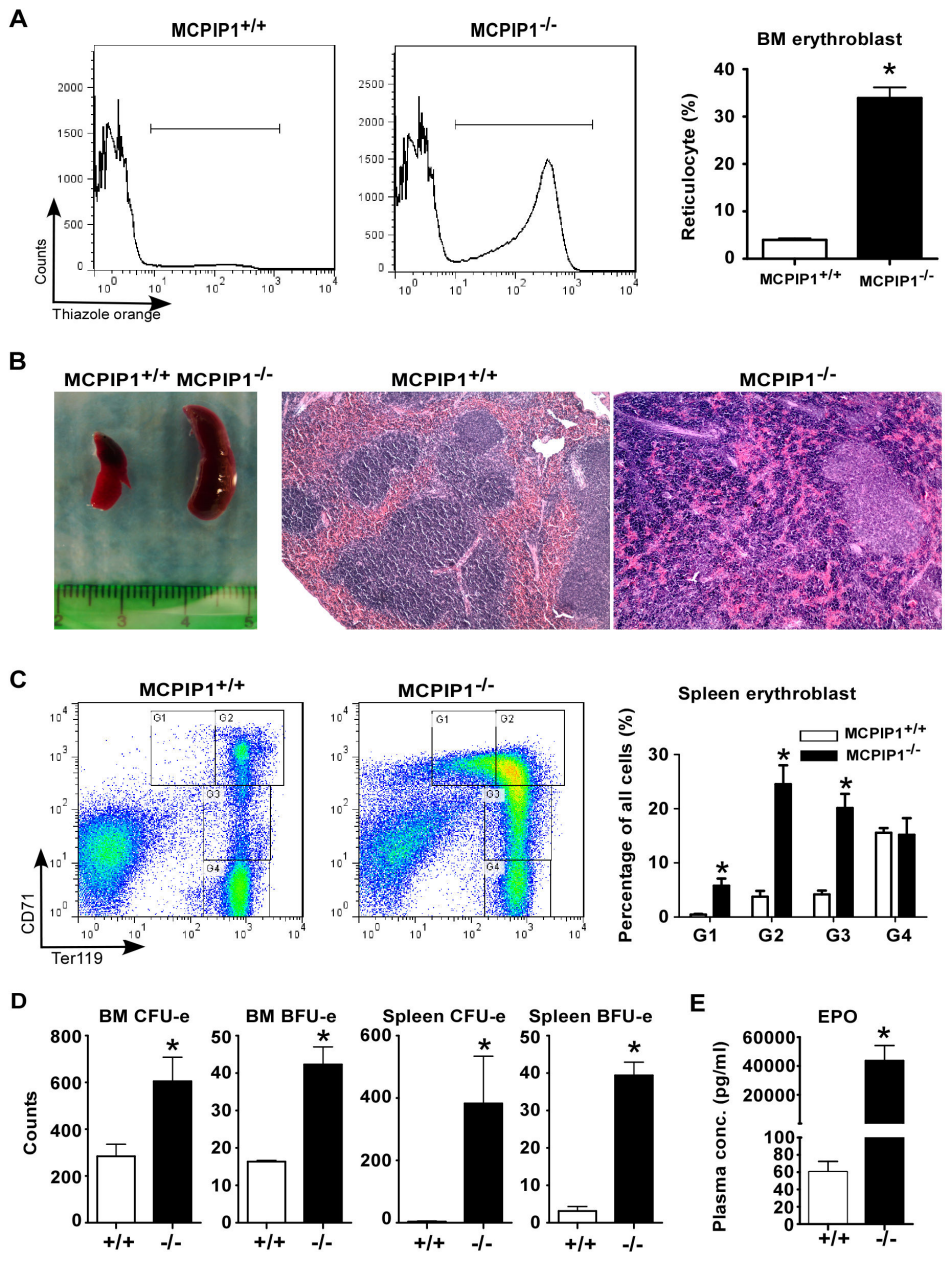
MCPIP1 deficiency did not compromise erythropoiesis *per* *se*. Reticulocyte percentage of the MCPIP1^+/+^ and MCPIP1^-/-^ peripheral blood was analyzed with flow cytometry (A). Spleens of these mice were shown and stained with H.E. (B). The splenocytes were also analyzed with Ter119/CD71 staining and the erythroblasts were gated from G1 to G4 (C). The bone marrow cells and splenocytes were also cultured *in* *vitro* to analyze the colony formation of CFU-es and BFU-es (D). MCPIP1^+/+^ and MCPIP^-/-^ plasma EPO concentration was analyzed with ELISA (E). N=4~6, *P<0.05.

 Because MCPIP1^-/-^ erythroblasts showed limited viability and proliferation *in vivo*, we suspected that these mice might have some degree of aplastic anemia, in which the erythropoietic potential is decreased. However, the peripheral blood thiazole orange^+^ reticulocyte percentage was dramatically higher in MCPIP1^-/-^ mice (Figure 2A), indicating an overall increased RBC production compensated with extramedullary hematopoiesis. Given spleen is a common compensatory hematopoietic organ in anemia condition and previous reports already found enlarged spleens in MCPIP1^-/-^ mice, we further examined the possible RBC production in spleens. In line with the enlarged spleens found in the MCPIP1^-/-^ mice, H.E. staining also showed expanded red pulp in MCPIP1^-/-^ spleens (Figure 2B), which is a common feature of spleen production of RBCs. More importantly, flow cytometry analysis revealed a drastic increase in G1~G3 erythroblast percentage in MCPIP1^-/-^ spleens (Figure 2C). These results suggested that in compensating the anemia condition, MCPIP1^-/-^ mice robustly produced more RBCs, part of which is from the extramedullary hematopoiesis in spleens. 

 To further examine the erythropoietic capability of BM and spleens of MCPIP1^-/-^ mice, we performed the *in vitro* colony formation assay. As expected, MCPIP1^-/-^ spleen cells produced dramatically more CFU-es and BFU-es (Figure 2D), suggesting that MCPIP1^-/-^ spleen can normally produce RBCs. Surprisingly, MCPIP1^-/-^ BM cells also gave rise to more RBC clone CFU-es and BFU-es than control BM cells (Figure 2D). These results suggested that both MCPIP1^-/-^ BM and spleen cells had improved RBC producing capacity. This is consistent with the enormously elevated plasma concentration of erythropoietin (EPO) (Figure 2E). 

 The results above showed reduced viability and proliferation of MCPIP1^-/-^ BM cells *in vivo*, but enhanced RBC colony formation *in vitro*. This difference indicated that the RBC production ability was not compromised in MCPIP1^-/-^ hematopoietic stem cells, but the RBC producing process was interfered *in vivo*, probably because of the lack of necessary nutrient components in erythropoiesis. 

### MCPIP1^-/-^ mice suffered from iron deficiency

**Figure 3:**
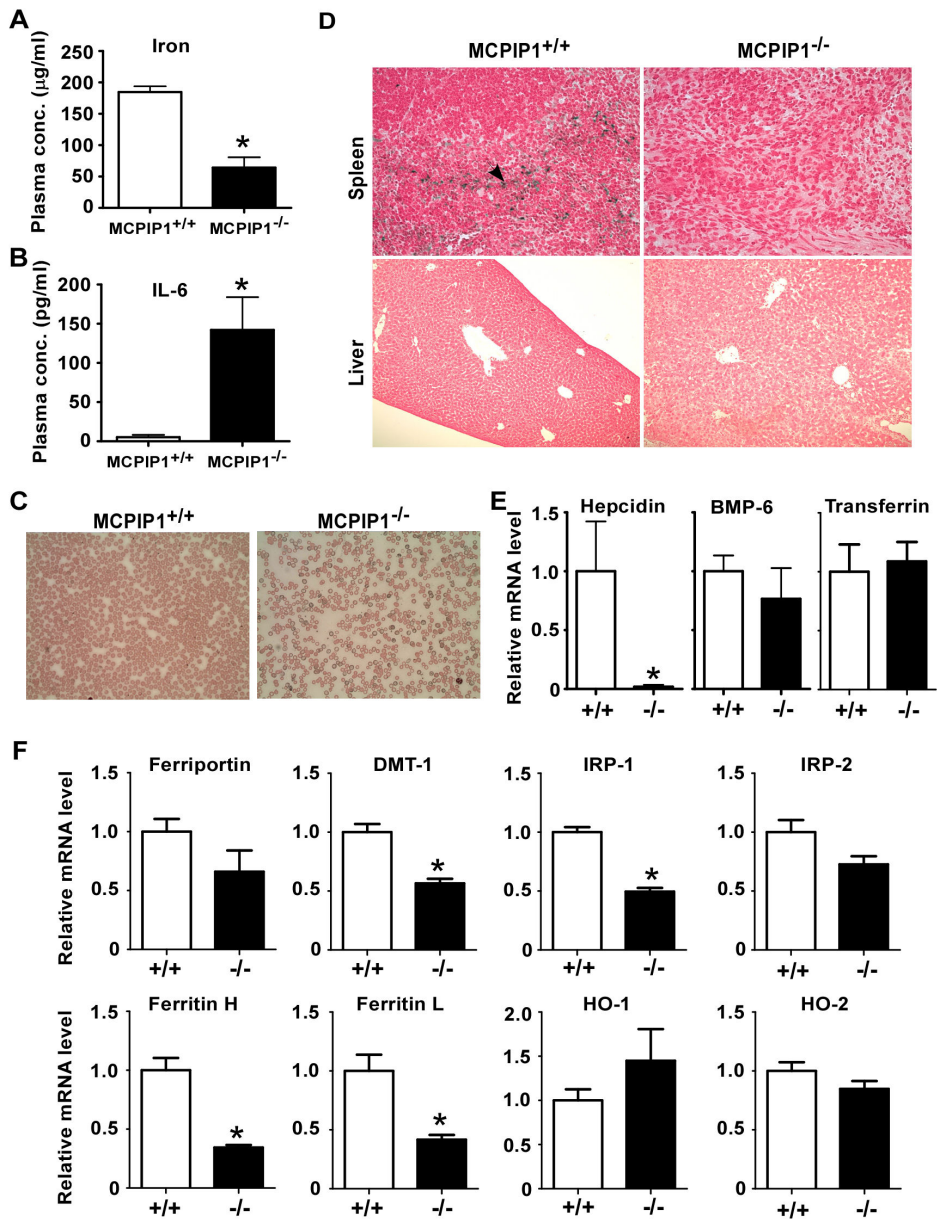
Iron deficiency in MCPIP1^-/-^ mice. Iron (A) and IL-6 (B) concentration in the MCPIP1^+/+^ and MCPIP1^-/-^ mice plasma was tested. The peripheral blood smear was also analyzed with Giemsa-Wright staining (C). The spleens and livers of MCPIP1^+/+^ and MCPIP1^-/-^ mice were examined with Prussian blue staining. Arrow indicated iron condensation (D). Liver (E) and intestine (F) iron metabolism related genes mRNA level was analyzed with real-time PCR. N=5, *P<0.05.

 One of the essential nutrients in erythropoiesis is iron. Chronic inflammation can cause iron re-distribution and consequently anemia. MCPIP1^-/-^ mice developed systemic inflammation and increased production of IL-6 [9,10]. We observed that MCPIP1^-/-^ mice had dramatically reduced iron plasma concentration (Figure 3A), and elevated IL-6 level (Figure 3B). The peripheral blood smear showed many red blood cells with less hemoglobin content (Figure 3C). These results suggested that MCPIP1^-/-^ mice had iron deficiency, which probably caused the anemia. However, is the iron deficiency caused by chronic inflammation? Hepcidin is the only hormone that mediates chronic inflammation-caused iron re-distribution and consequent anemia of chronic inflammation. Although plasma IL-6 level was higher in MCPIP1^-/-^ mice, to our surprise, the hepcidin mRNA level was drastically lower (Figure 3E), and the iron condensation in livers and spleens was totally absent in MCPIP1^-/-^ mice, despite the normal iron storage shown in MCPIP1^+/+^ spleens (Figure 3D). Moreover, the expression of bone morphogenetic protein-6 (BMP-6), a secondary mediator of chronic inflammation anemia, did not exhibit any change, neither did the major iron carrying protein produced by the livers, transferrin (Figure 3E). Thus, the iron deficiency was not caused by chronic inflammation. We reasoned that the iron overall uptake rather than its distribution in MCPIP1^-/-^ mice was compromised. We next analyzed the expression level of the critical genes related with iron absorption in intestine. Although divalent metal transporter-1 (DMT-1) expression was reduced in MCPIP1^-/-^ intestine, iron regulatory protein-1 (IRP-1), Ferritin H and L were also down-regulated in compensation (Figure 3F). In addition, intestinal bleeding and immunoglobin condensation were not observed in MCPIP1^-/-^ mice (data not shown). Taken together, these results suggest that the iron deficiency was not caused by chronic inflammation and iron re-distribution, but may be caused by abnormal iron absorption other than intestine dysfunction. 

### MCPIP1^-/-^ mice had autoimmune gastritis, parietal cell loss and VB_12_ deficiency

**Figure 4:**
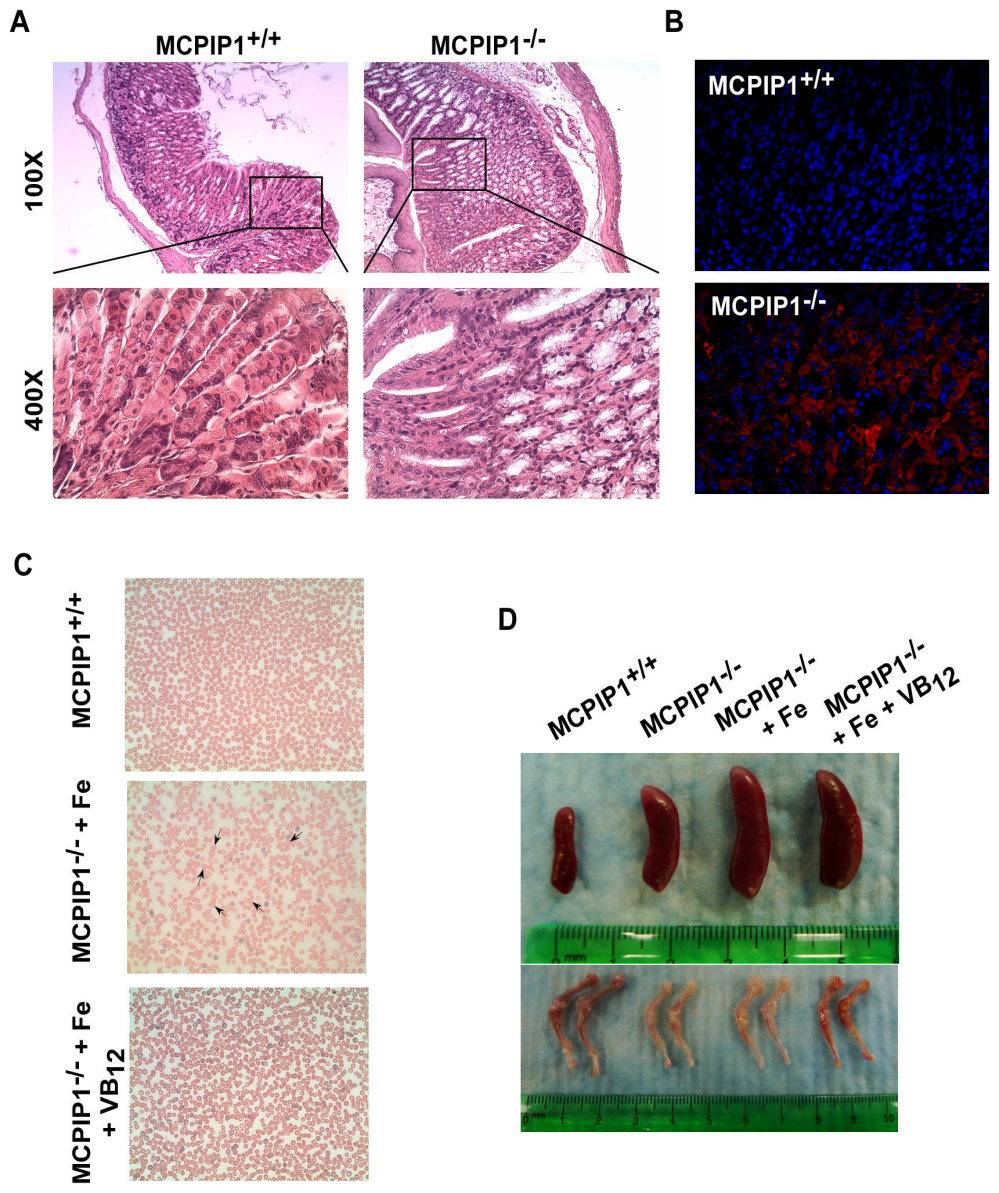
MCPIP1^-/-^ mice developed autoimmune gastritis, parietal cell loss and VB_12_ deficiency. The stomach mucosa of MCPIP1^+/+^ and MCPIP1^-/-^ mice was performed with H.E. staining (A) and immunofluorescent staining of IgG (red) and DAPI (blue) (400×, B). The 6 weeks old MCPIP1^-/-^ mice were supplemented with iron dextrin with or without VB_12_. 7 days later the peripheral blood smear Giemsa-Wright staining was performed (C, arrowheads indicate the megaloblastic RBCs found only in the iron supplementation group) and the spleens, femurs and tibias were shown (D). Data represent 1 of at least 3 independent experiments.

 It is well-known that gastric acid secretion from parietal cells in the stomach is necessary for sufficient iron absorption [5]. Thus, we analyzed whether the parietal cells in MCPIP1^-/-^ mice were intact. We observed that stomachs from MCPIP1^-/-^ mice were shrunken (data not shown), and there was a dramatic loss of parietal cells in the stomach mucosa (Figure 4A). We further examined the autoimmune antibodies in the stomach, and found significant condensation of IgG in MCPIP1^-/-^ stomach mucosal areas, but not in MCPIP1^+/+^ littermate controls (Figure 4B). Thus, MCPIP1^-/-^ mice suffered from autoimmune gastritis and parietal cell loss. 

**Figure 5:**
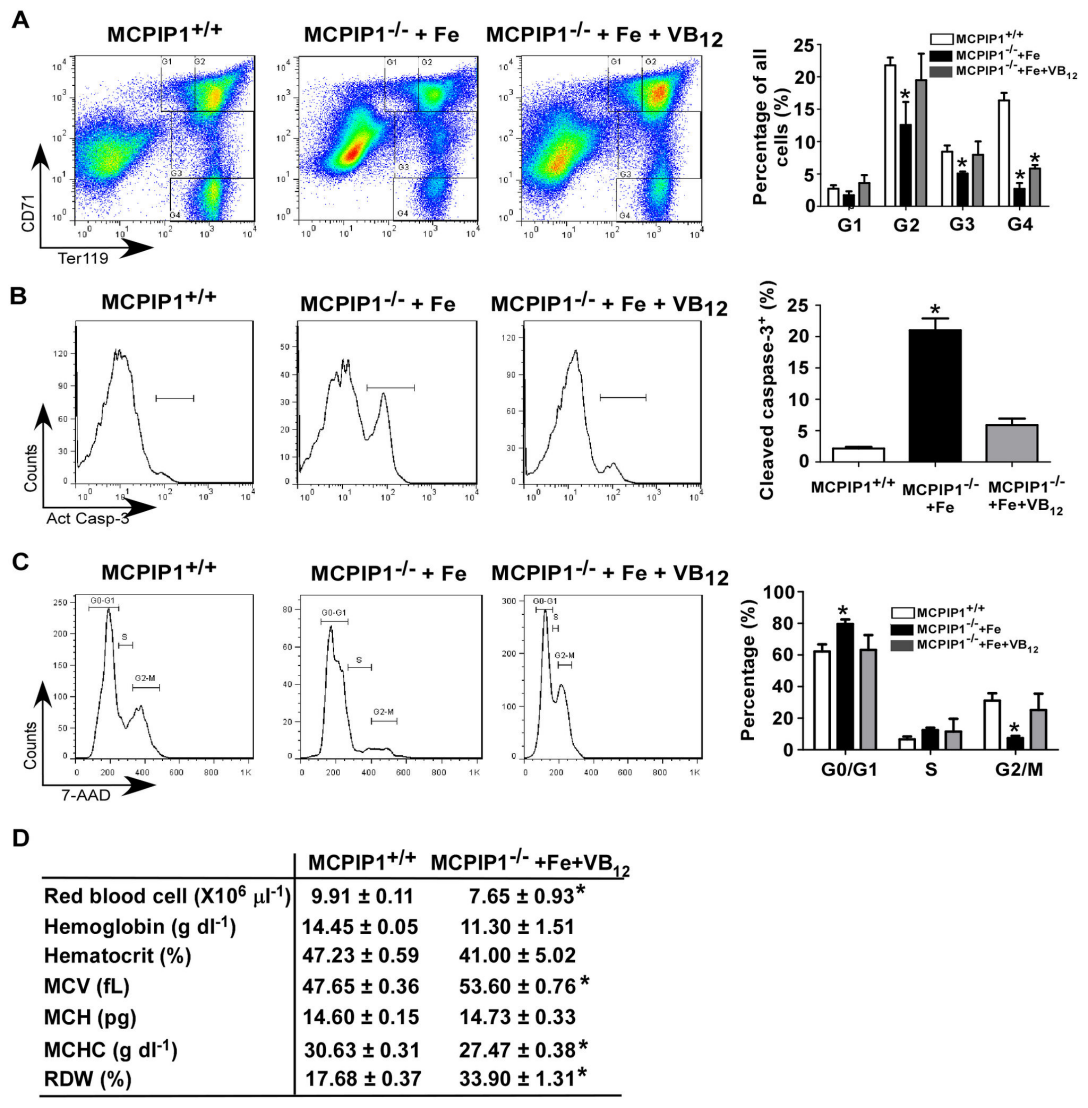
Supplementation with iron and VB_12_ greatly improved anemia in MCPIP1^-/-^ mice. MCPIP1^-/-^ mice were treated with iron or combined iron plus VB_12_ for 7 days. Then, the bone marrow erythroblast Ter119/CD71 gating (A), active caspase-3 staining (B) and cell cycle analysis (C) were performed. The peripheral red blood cell count was also performed on the iron and VB_12_ treated MCPIP1^-/-^ mice compared with the MCPIP1^+/+^ ones (D). N=3~4, *P<0.05 versus MCPIP1^+/+^ group.

 Previous studies found that parietal cells are critically involved in VB_12_ absorption by secreting intrinsic factor, and the loss of parietal cells is usually the reason of megaloblastic pernicious anemia [4,11]. Because the parietal cell loss in our study was similar to previous pernicious anemia reports [12], we suspected that MCPIP1^-/-^ mice might also have VB_12_ deficiency and pernicious anemia. So we examined whether supplementation with iron or iron plus VB_12_ could rescue the anemia phenotype of MCPIP1^-/-^ mice. After 1 week of injection, MCPIP1^-/-^ mice treated with iron showed megaloblastic RBCs in the peripheral blood (Figure 4C), which further confirmed the VB_12_ deficiency phenotype. Moreover, MCPIP1^-/-^ mice received iron and VB_12_ showed similar RBC shape with MCPIP1^+/+^ controls (Figure 4C). The bone marrow from the iron and VB_12_ treated mice showed comparable color with MCPIP1^+/+^ mice, while the iron alone treated bone marrow was not (Figure 4D). More importantly, the iron plus VB_12_ supplementation greatly improved the bone marrow G1 and G2 erythroblast percentage, the erythroblast apoptosis and proliferation (Figure 5 A~C), Iron and VB_12_ combined supplementation greatly rescued the anemia phenotype in MCPIP1^-/-^ mice (Figure 5D). Taken together, these results suggest that the anemia phenotype of MCPIP1^-/-^ mice may be caused by autoimmune gastritis, parietal cell loss and consequently iron and VB_12_ deficiency. 

### MCPIP1^-/-^ mice developed autoimmune antibodies against RBCs

**Figure 6:**
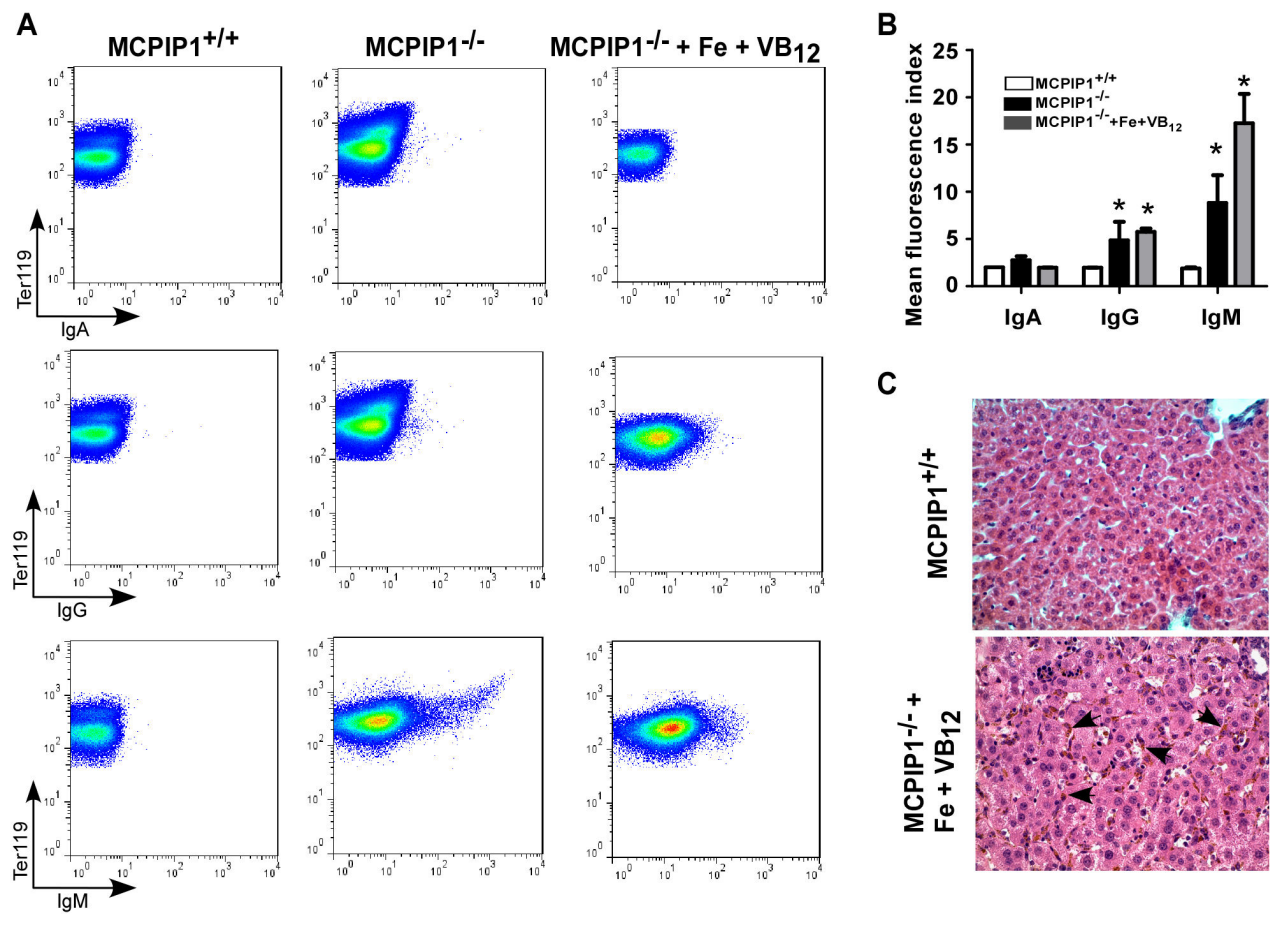
Increased immunoglobin attack and macrophage engulfment of RBCs in MCPIP1^-/-^ mice. The peripheral blood cells were stained with Ter119 to define RBCs and IgA, IgG and IgM Fc antibodies in MCPIP1^+/+^ and MCPIP1^-/-^ mice treated with or without iron plus VB_12_ (A). The mean fluorescence index in A was further statistically analyzed (B). The livers in MCPIP1^+/+^ and MCPIP1^-/-^ mice treated with iron plus VB_12_ were H.E. stained and the arrowheads point out the hemosiderin in the livers (C). In A and B, N=3~6, *P<0.05 versus MCPIP1^+/+^ group. C represents one of four independent experiments.

 As the supplementation of VB_12_ and iron did not totally rescue the anemia phenotype of MCPIP1-/- mice, we wondered whether RBCs are directly attacked by autoimmune antibodies in the MCPIP1^-/-^ mice. Thus, we analyzed the antibody attachment of RBCs in the peripheral blood of MCPIP1^-/-^ mice. We found that the RBCs were attacked with IgG and IgM, with higher level on the latter (Figure 6A and B). RBC autoreactive IgM antibodies can damage erythrocytes through macrophages by removal of these tagged erythrocytes from circulation [13]. We next examined whether the degradation of erythrocytes was increased in MCPIP1^-/-^ livers. As shown in Figure 6C, hemosiderin deposition, indicating erythrocyte clearance, was greatly increased in livers of MCPIP1^-/-^ mice. These results suggested that the RBCs are directly attacked by autoimmune IgG and IgM, resulting in their accelerated degradation by macrophages. This autoimmunity against RBCs might be another mechanism that the MCPIP1^-/-^ mice had anemia in addition to the parietal cell autoimmune damage. 

## Discussion

 Anemia is a common disease of RBC deficiency. Previous reports described an anemia phenotype in MCPIP1^-/-^ mice. However, the underlying mechanisms have not been reported. In the present study, we found that MCPIP1 deficiency in mice resulted in severe anemia related to autoimmune mechanisms. MCPIP1 deficiency did not affect erythropoiesis *per se*. In contrast, the MCPIP1^-/-^ mice developed autoimmune gastritis and parietal cell loss. The consequent malfunction of iron and VB_12_ absorption finally lead to pernicious anemia. Thus, the MCPIP1^-/-^ mice might be a model for the study of autoimmune gastritis against parietal cells, and pernicious anemia. In addition, MCPIP1 deficiency also led to the production of anti-RBC autoimmune antibody and probable hemolytic anemia. Our present work provided evidence of an immune regulatory role of MCPIP1 and the significance of preventing autoimmunity related anemia. 

 Although MCPIP1^-/-^ mice had anemia, their hematopoietic activity was not compromised. These mice had elevated EPO plasma concentration and higher peripheral reticulocyte count. The *in vitro* colony formation assays also showed more abundant CFU-es and BFU-es from MCPIP1^-/-^ bone marrow and spleens. These results are consistent with an increased rather than decreased hematopoietic potency [1,14,15], and suggest that the anemia phenotype of MCPIP1^-/-^ mice was not aplastic anemia, but may be resulted from extrinsic abnormalities of erythropoiesis. Indeed, MCPIP1^-/-^ mice had lower plasma iron concentration and the peripheral blood smear showed massive hypochromic RBCs, which was a feature of iron deficiency caused hemoglobin reduction [6]. Although MCPIP1^-/-^ mice developed systemic inflammation, the chronic inflammation is not the reason of iron deficiency, as the liver hepcidin mRNA was not increased but decreased in MCPIP1^-/-^ mice and the re-distribution of iron was not observed. Thus, the iron deficiency in MCPIP1^-/-^ mice was not a result of global inflammation, but may be a result of the abnormal absorption of iron. 

 Parietal cells are a type of epithelial cells in the stomach mucosa. The two functions of parietal cells are secreting gastric acid [16] and producing intrinsic factor [4], which are critical for VB_12_ as well as iron absorption. In the present study we found series of evidence of pernicious anemia in MCPIP1^-/-^ mice, including parietal cell loss[4,11], autoimmune IgG condensation in the stomach mucosa[11] and enlarged mature RBCs[4]. The increased apoptosis and decreased proliferation in bone marrow erythroblast can also attribute to VB_12_ deficiency. VB_12_ is a necessary material for thymidine production and DNA replication. The lack of VB_12_ would result in delayed cell cycle and DNA breakage, and finally erythroblast cell death [17]. More importantly, the supplementation of VB_12_ and iron rescued the anemia phenotype in MCPIP1^-/-^ mice, while single iron injection did not, which further confirmed the pernicious anemia and the importance of VB_12_ deficiency in MCPIP1^-/-^ mice. In addition, as VB_12_ and iron supplementation did not completely rescue the anemia in MCPIP1^-/-^ mice, other mechanisms may also exist. We found some evidence indicating that autoimmune hemolysis may also contribute to the anemia phenotype of MCPIP1^-/-^ mice. The autoimmunity against RBCs may unveil its significance especially when the iron and VB_12_ are supplemented. The correction of the nutrition deficiency would provide sufficient oxygen to the autoimmune lymphocytes and the surplus VB_12_ would facilitate the autoimmune lymphocyte proliferation, both of which would exacerbate the RBC autoimmunity [18]. Thus, after supplementation of iron and VB_12_, the MCPIP1^-/-^ mice may also be adequate to be used in the research of autoimmune hemolytic anemia.

## Conclusions

 We are here reporting the underlying mechanisms of the anemia phenotype of MCPIP1^-/-^ mice. We provided evidence that the anemia phenotype of MCPIP1^-/-^ mice was caused by iron deficiency, VB_12_ deficiency and RBC rapid clearance, which was mainly resulted from autoimmune-associated gastritis and hemolysis. MCPIP1^-/-^ mice may be a good mouse model for investigating the pathogenesis and possible treatment of autoimmune gastritis and pernicious anemia. 
